# Citrate pharmacokinetics in critically ill liver failure patients receiving CRRT

**DOI:** 10.1038/s41598-022-05867-8

**Published:** 2022-02-02

**Authors:** Peerapat Thanapongsatorn, Weerachai Chaijamorn, Phatadon Sirivongrangson, Sasipha Tachaboon, Sadudee Peerapornratana, Nuttha Lumlertgul, Aroonrut Lucksiri, Nattachai Srisawat

**Affiliations:** 1 Central Chest Institute of Thailand, Nonthaburi, Thailand; 2 Excellence Center for Critical Care Nephrology, King Chulalongkorn Memorial Hospital, Bangkok, Thailand; 3 Division of Nephrology, Department of Medicine, Critical Care Nephrology Research Unit, Faculty of Medicine, Chulalongkorn University, Bangkok, 10330 Thailand; 4 Faculty of Pharmacy, Siam University, Bangkok, Thailand; 5 Department of Pharmaceutical Care, Faculty of Pharmacy, Chiang Mai University, Chiang Mai, Thailand; 6 Academy of Science, Royal Society of Thailand, Bangkok, Thailand; 7 Department of Medicine, Somdech Phra Pinklao Hospital, Bangkok, Thailand; 8 Center of Excellence in Critical Care Nephrology, Chulalongkorn University, Bangkok, 10330 Thailand

**Keywords:** Continuous renal replacement therapy, Pharmacokinetics, Liver cirrhosis

## Abstract

Citrate has been proposed as anticoagulation of choice in continuous renal replacement therapy (CRRT). However, little is known about the pharmacokinetics (PK) and metabolism of citrate in liver failure patients who require CRRT with regional citrate anticoagulation (RCA). This prospective clinical PK study was conducted at King Chulalongkorn Memorial Hospital between July 2019 to April 2021, evaluating seven acute liver failure (ALF) and seven acute-on-chronic liver failure (ACLF) patients who received CRRT support utilizing RCA as an anticoagulant at a citrate dose of 3 mmol/L. For evaluation of the citrate PK, we delivered citrate for 120 min and then stopped for a further 120 min. Total body clearance of citrate was 152.5 ± 50.9 and 195.6 ± 174.3 mL/min in ALF and ACLF, respectively. The ionized calcium, ionized magnesium, and pH slightly decreased after starting citrate infusion and gradually increased to baseline after stopping citrate infusion. Two of the ACLF patients displayed citrate toxicity during citrate infusion, while, no ALF patient had citrate toxicity. In summary, citrate clearance was significantly decreased in critically ill ALF and ACLF patients receiving CRRT. Citrate use as an anticoagulation in these patients is of concern for the risk of citrate toxicity.

## Introduction

Regional citrate anticoagulation (RCA) is the first-line anticoagulation in critically ill patients receiving continuous renal replacement therapy (CRRT)^1^. Many studies have shown an impressive benefit of RCA to extend circuit lifespans, reduce the incidence of hemorrhagic complications, and lower transfusion needs^2–6^. Citrate, the anionic salt of citric acid, anticoagulated the extracorporeal circuit by chelating ionized calcium (Ca^2+^), which is a key cofactor in the clotting cascade^7,8^. Under physiological conditions, citrate is mainly metabolized by mitochondria in the liver, skeletal muscle, and the kidney into bicarbonate within 5 min^9,10^. Therefore, in the setting of acute liver failure (ALF) or acute-on-chronic liver failure (ACLF), citrate metabolism may be incomplete and lead to citrate toxicity. The toxicity of citrate during CRRT is defined as citrate accumulation and presents as low Ca^2+^ concentrations, due to the complex binding of citrate and Ca^2+^ and a need of calcium substitution; metabolic acidosis, due to the decreased citric acid cycle production of bicarbonate; and a total calcium (Ca_tot_)/Ca^2+^ ratio of > 2.5^11,12^.

With respect to citrate pharmacokinetics (PK) in critically ill patients with acute kidney injury (AKI), it was reported that citrate clearance is not impaired in AKI patients^13^. In contrast, the citrate PK in critically ill patients with cirrhosis was impaired by 50% compared to non-cirrhotic critically ill patients^14^. To the best of our knowledge, no study has been conducted to demonstrate the citrate PK in critically ill ALF or ACLF patients receiving CRRT.

Therefore, to address this knowledge gap of RCA in CRRT, we first evaluated the citrate PK and metabolism among critically ill acute decompensated liver failure patients receiving CRRT.

## Material and methods

### Trial design and oversight

This study was a prospective clinical PK study at King Chulalongkorn Memorial Hospital (KCMH) in Thailand from July 2019 to April 2021. The trial was retrospective registered at Thai Clinical Trials Registry on 07/07/2021 (TCTR20210707010) and was approved by the institutional review board of the Faculty of Medicine, Chulalongkorn University, Bangkok, Thailand (IRB No. 694/63). The study was conducted according to the Declaration of Helsinki and Good Clinical Practice guidelines, and all study methods were carried out in accordance with relevant guidelines and regulations. Written informed consent was obtained from all patients by their close relatives or themselves (if eligible) before enrollment. All potential patients who declined to participate were received the standard treatment included CRRT, vasopressor, appropriate antibiotics, etc. for their conditions and were not disadvantaged by not participating for the study.

### Patients

The inclusion criteria were critically ill ALF or ACLF adult patients with AKI, aged ≥ 18 years old, and receiving CRRT. The ALF was defined as an acute onset of severe liver injury with (i) a worsening liver function test with abnormal coagulation, usually with an INR ≥ 1.5, and (ii) any degree of hepatic encephalopathy in a patient without pre-existing liver disease, according to The Asian Pacific Association for the Study of the Liver (APASL) consensus 2014. Whereas, ACLF was defined as ALF in a patient with a pre-existing liver disease confirmed by ultrasonography or liver biopsy^15,16^. The exclusion criteria were severe acidosis (pH < 7.1) or severe alkalosis (pH > 7.55), blood transfusion within 24 h prior to the study, use of citrate-containing medications, severe hypocalcemia (serum ionized calcium < 0.8 mmol/L), and use of heparin as anticoagulation.

### Study protocol

For the citrate PK study, CRRT was performed as continuous venovenous hemofiltration (CVVH) mode with 100% pre-dilution, using the PRISMAFLEX system version 8.1 (Baxter Healthcare /Gambro Lundia AB, Sweden) with a standard PRISMA M-100 AN69 (polyacrilonitrile) hemofilter. The pre-dilution replacement solution was infused via pre-blood pump line (white pump) of PRISMAFLEX system. Citra-HF-Pre (Nordic Medcom, Finland) was used as pre-dilution replacement solution for isotonic citrate solution (consisted of citrate 13.3 mmol/L, Na^+^ 139.9 mmol/L, K^+^ 3 mmol/L, Mg^2+^ 0.5 mmol/L, and Cl^−^ 104 mmol/L) for 120 min. The pre-dilution replacement flow rate and blood flow rate was calculated to achieve the prescribed pre-filter citrate dose of 3 mmol/L and the effluent dose of 25–30 mL/kg/h. A pre-dilution replacement flow rates in weight ranges (< 60, 60–70, and > 70 kg) to match the prespecified citrate dose were provided in the Supplementary Table 1. The ultrafiltration (UF) rates were adjusted based on the patient’s fluid status. However, the UF rates were not permitted to exceed a net negative balance of 400 mL per 3 h.

To prevent hypocalcemia, we used a constant rate of 10% calcium gluconate (0.225 mmol/mL) infused into the venous return line during citrate infusion. After 120 min, the isotonic citrate solution was stopped and switched to non-citrated isotonic solution using PRISMASOL B0 (Baxter, Italy), which consisted of Na^+^ 140 mmol/L, Ca^2+^ 1.75 mmol/L, Mg^2+^ 0.5 mmol/L, and Cl^−^ 109.5 mmol/L. The concentration of KCl in the replacement fluid was 3 mmol/L, the same as the concentration in Citra-HF-Pre. The calcium gluconate infusion was also stopped after citrate infusion ended. The detail of the CRRT protocol and calcium infusion rate are shown in Supplementary Table 1.

Arterial blood samples were collected at pre-filter at baseline and after 10, 20, 40, 60, 120 (citrate infusion stopped), 125, 130, 140, 160, 180, 210, and 240 min. In addition, 10 min after the end of citrate infusion, blood samples were taken simultaneously from both the pre-filter and post-filter to calculate the citrate clearance via the hemofilter (Supplementary Fig. 1). The blood samples were sent for determination of the citrate, blood gas, electrolyte, calcium, magnesium, Ca^2+^, and Mg^2+^ concentrations. The blood gas samples were collected in a lithium heparin syringe and then analyzed by a Nova Stat Profile Prime Plus® (Nova Biomedical, Waltham, MA, USA). The citrate concentrations were measured using a commercially available kit (BOEHRINGER MANNHEIM / R-BIOPHARM Enzymatic BioAnalysis Cat. No. 10 139 076 035).

For citrate toxicity monitoring, we observed citrate accumulation, which was defined as (i) a Ca_tot_/Ca^2+^ ratio of > 2.5, (ii) metabolic acidosis, and (iii) low Ca^2+^ level. The Ca_tot_/Ca^2+^ ratio was monitored every 120 min.

### Calculation of citrate PK

The PK parameters were performed via non-compartmental analysis using the Phoenix® WinNonlin® version 8.2 software program (Certara USA, Inc., Princeton, NJ). The area under the plasma concentration–time curve (AUC) was calculated by employing the linear trapezoidal rule. The time to reach maximum concentration (T_max_) and the maximum citrate concentration (C_max_) were measured directly from the concentration–time curve.

The volume distribution (Vd) was calculated by the standard formula; Vd = Cl/k_e_, whereas k_e_ is the elimination rate constant and Cl (total drug clearance) was calculated by dose/AUC_0–inf_. The body citrate clearance was calculated by subtraction of the filter citrate clearance (Cl_filter_) from the total citrate clearance, where Cl_filter_ was calculated based on the equations from Zheng and colleagues study^13^, as shown in Eq. (1);

1 $${\text{Cl}}_{{{\text{filter}}}} = \frac{{Qpw * Cin {-} \left( {Qpw - UF} \right)* Cout}}{Cin}$$

where *Cin* and *Cout* are the plasma water concentrations of citrate at pre-filter (in) and post-filter (out), respectively. The blood flow rate (*Qpw*) was calculated from Eq. (2),

2 $$Qpw = {\text{blood }}\;{\text{flow }}\;{\text{rate}}* \, ({1} - {\text{hematocrit}}\left( \% \right)) \, *{1} - \, \left( {\frac{{{\text{total}} \, {\text{ protein }} \, {\text{concentration}}}}{100}} \right)$$

### Data analysis

Continuous variables are presented as the mean ± standard deviation (SD) in case of a normal distribution, and as a median and interquartile range (IQR) in case of non-normally distributed variables. Categorical variables are characterized by numbers with percentages. All statistical analyses were performed using the SPSS Version 22 software (SPSS, Chicago, IL), and figures were drawn using GraphPad Prism 8 (GraphPad Software Inc., California, USA).

### Ethics approval and consent to participate

This study was conducted according to the Declaration of Helsinki and Good Clinical Practice guidelines. The study protocol was approved by the institutional review board of the Faculty of Medicine, Chulalongkorn University, Bangkok, Thailand (IRB No. 694/63). Written informed consent was obtained from all patients by their close relatives or themselves (if eligible) before enrollment.

## Results

### Patient characteristics

The patients’ baseline characteristics are summarized in Table 1. The causes of ALF in the ALF group were ischemic hepatitis (n = 4) and drug induced hepatitis (n = 3), whereas in the ACLF group they were ischemic hepatitis (n = 6) and autoimmune hepatitis (n = 1). The causes of cirrhosis in the ACLF group were viral hepatitis (n = 2), non-alcoholic steatohepatitis (NASH) (n = 2), alcohol, autoimmune hepatitis, and cardiac cirrhosis (n = 1 each). The mean APACHE II and SOFA scores were lower in the ALF group (19.6 ± 4.1 and 13.7 ± 3.7, respectively), than in the ACLF group (24.0 ± 5.6 and 15.6 ± 3.5, respectively).

**Table 1:** Baseline characteristics.

Patient	Age/sex	Admission diagnosis	Cause of AKI	Cause of cirrhosis	Cause of ALF	APACHE II score	SOFA score	Survival status
1	47/M	Post heart transplantation	Cardiorenal syndrome	None	Ischemic hepatitis	16	16	Alive
2	67/F	Scleroderma renal crisis	Sepsis AKI	None	Ischemic hepatitis	19	18	Death
3	53/M	Chikungunya viral infection	Sepsis AKI	None	Ischemic hepatitis	16	13	Alive
4	68/M	Upper GI bleeding	Ischemic ATN	None	Ischemic hepatitis	18	12	Death
5	57/M	Pneumonia with septic shock	Sepsis AKI	None	Drug induced hepatitis	21	7	Death
6	46/M	Disseminated TB	Sepsis AKI	None	Drug induced hepatitis	28	17	Death
7	81/M	Drug induced hepatitis	Sepsis AKI	None	Drug induced hepatitis	19	13	Death
8	52/M	Massive upper GI bleeding	Hepatorenal syndrome	Alcoholic	Ischemic	20	16	Death
9	68/F	Cryoglobulinemia	Septic AKI	HBV	Ischemic	30	19	Death
10	53/F	PCP pneumonia	Septic AKI	Autoimmune hepatitis	Autoimmune	15	15	Death
11	97/M	Pneumonia with septic shock	Septic AKI	NASH	Ischemic	23	19	Death
12	85/M	Cardiogenic shock	Cardiorenal syndrome	Cardiac cirrhosis	Ischemic	22	11	Death
13	43/M	Pneumonia with septic shock	Septic AKI	HCV	Ischemic	30	11	Death
14	79/M	Primary bacteremia with septic shock	Septic AKI	NASH	Ischemic	28	18	Death

*AKI* acute kidney injury, *APACHE II score* Acute Physiology and Chronic Health Evaluation II score, *GI* gastrointestinal, *HBV* hepatitis B virus, *HCV* hepatitis C virus, *NASH* non-alcoholic steatohepatitis; PCP, pneumocystis pneumonia, *SOFA score* Sequential Organ Failure Assessment score, *TB* tuberculosis.

The baseline laboratory data of the two groups are summarized in Table 2. Most laboratory values were comparable between the two groups, except for the direct bilirubin values, which were higher in the ALF group than in the ACLF group (11.3 [9.8,14.5] vs. 6.2 [3.6,11.2] mg/dL).

**Table 2:** Baseline laboratory data.

	Critically ill ALF patients (N = 7)	Critically ill ACLF patients (N = 7)
Hemoglobin (g/dL)	9.1 ± 1.4	9.0 ± 2.2
White blood cells (× 10^3^/µL)	12.6 ± 8.2	23.5 ± 11.0
Platelets (× 10^3^/µL)	55.4 ± 33.1	100.4 ± 55.3
BUN (mg/dL)	30.6 ± 24.8	27.9 ± 11.8
Cr (mg/L)	1.5 ± 1.2	1.8 ± 1.0
Sodium (mmol/L)	135 ± 6	136 ± 5
Potassium (mmol**/**L)	3.7 ± 0.5	4.2 ± 0.8
Bicarbonate (mmol**/**L)	22.3 ± 2.9	21.7 ± 5.2
Calcium (mg**/**dL)	8.8 ± 0.9	8.3 ± 1.6
Magnesium (mg**/**dL)	0.9 ± 0.1	0.9 ± 0.1
Phosphate (mg**/**dL)	2.3 ± 0.9	4.1 ± 2.5
Total bilirubin (mg**/**dL)	19.5 ± 8.9	11.1 ± 5.1
Direct bilirubin (mg**/**dL)	11.3 (9.8,14.5)	6.2 (3.6, 11.2)
AST (U**/**L)	128 (50,355)	165 (58,614)
ALT (U**/**L)	120 (23,352)	112 (21,224)
ALP (U**/**L)	130 (78,170)	165 (99,183)
Albumin (g**/**L)	3.2 ± 0.7	3.3 ± 0.5
PT (s)	21.2 ± 8.8	27.4 ± 15.2
INR	1.9 (1.4, 2.6)	1.8 (1.6, 3.9)
aPTT (s)	36.3 (27.7, 52.0)	51.5 (34.5, 84.5)
pH	7.42 ± 0.06	7.39 ± 0.08
Lactate (mmol**/**L)	3.26 ± 2.83	5.47 ± 2.00
Ionized calcium (mmol**/**L)	1.18 ± 0.10	1.08 ± 0.22
Ionized magnesium (mmol**/**L)	0.59 ± 0.11	0.51 ± 0.07

*ALT* alanine aminotransferase, *ALP* alkaline phosphatase, *aPTT* activated partial thromboplastin time, *AST* aspartate aminotransferase, *BUN* blood urea concentration, *Cr* creatinine, *INR* international normalized ratio, *PT* prothrombin time.

Data are represented as mean ± SD or median (IQR).

### Citrate PK

**Figure 1:**
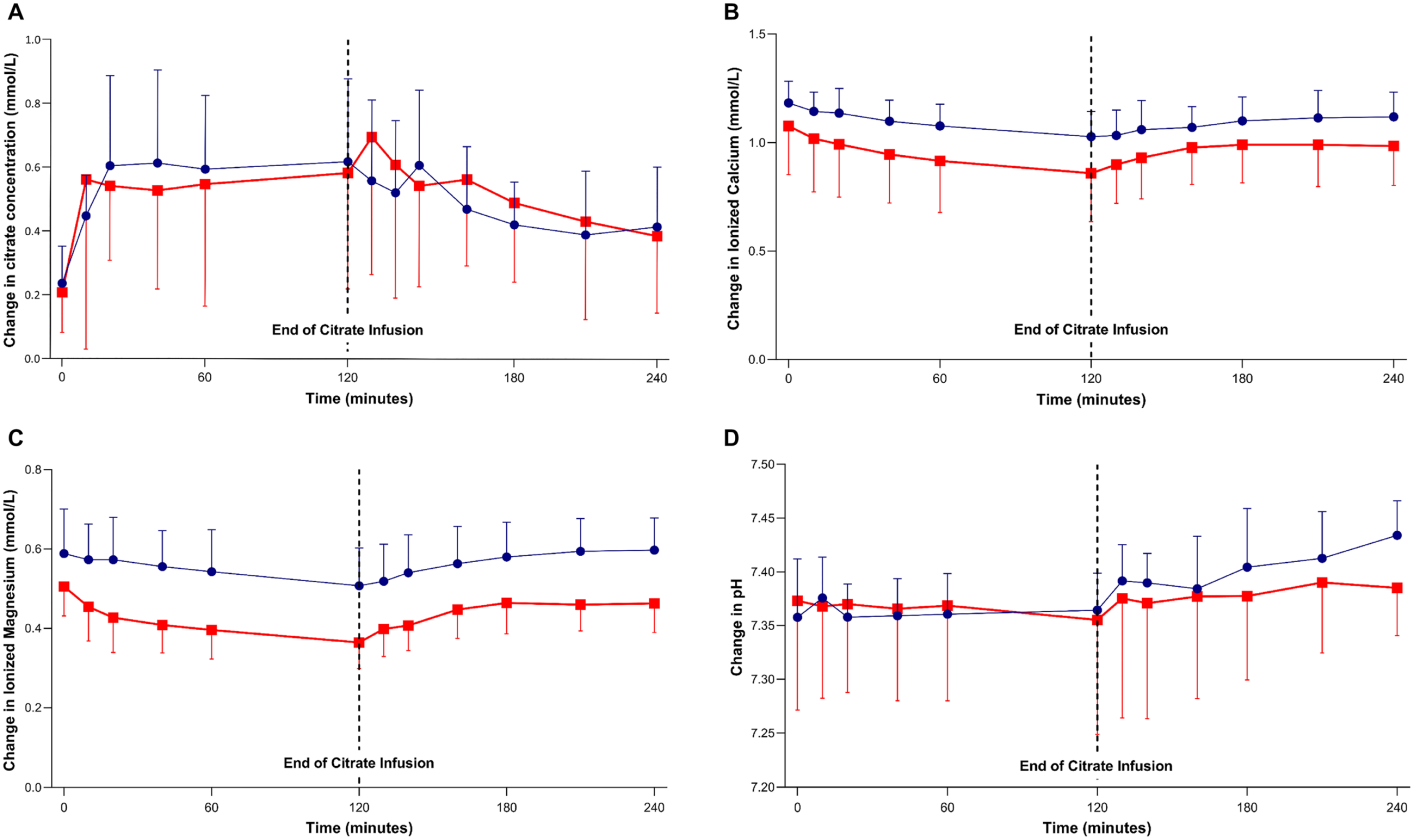
Comparison of ALF (blue circles) and ACLF (red squares). The results for the plasma citrate concentration (**A**), Ca^2+^ (**B**), Mg^2+^ (**C**), and blood pH (**D**) during 120 min of citrate infusion and 120 min after citrate infusion was stopped. Data are presented as the mean ± 1SD.

The citrate PK results are shown in Table 3, while the average citrate concentrations during citrate infusion until 120 min after the infusion was stopped are illustrated in Fig. 1A. In ALF patients, the citrate concentration gradually increased from baseline during infusion to a maximum concentration of 0.76 ± 0.27 mmol/L after 100.0 ± 60.0 min. The average body citrate clearance was 152.5 ± 50.9 mL/min. Whereas, in the ACLF patients, the time to reached a broadly similar maximum citrate concentration (0.72 ± 0.44 mmol/L) was numerically longer at 113.8 ± 32.8 min, giving a numerically higher body citrate clearance rate (195.6 ± 174.3 mL/min). After stopping citrate infusion, the citrate concentration was consistently decreased in both groups.

**Table 3:** Citrate PK.

Parameter	Critically ill ALF patients (N = 7)	Critically ill ACLF patients (N = 7)
AUC_0−t_ (mmol min/L)	124.4 ± 43.9	113.9 ± 73.3
AUC_0−inf_ (mmol min/L)	267.2 ± 111.7	372.6 ± 399.3
T_max_ (min)	100.0 ± 60.0	113.8 ± 32.8
V_d_ (L)	45.6 ± 8.0	58.2 ± 49.7
Cl_body_ (mL/min)	152.5 ± 50.9	195.6 ± 174.3
C_baseline_ (mmol/L)	0.24 ± 0.12	0.21 ± 0.12
C_max_ (mmol/L)	0.76 ± 0.27	0.72 ± 0.44
Total dose (mmol)	39.9	39.9

*AUC* area under the plasma concentration–time curve, *T*_*max*_ time to maximum concentration, *V*_*d*_ volume of distribution, *Cl*_*body*_ citrate clearance by body, *C*_*baseline*_ baseline citrate concentration, *C*_*max*_ maximum citrate concentration.

Data are shown as mean ± SD.

### Change in the Ca^2+^ and Mg^2+^ concentrations

The Ca_tot_ and Ca^2+^ levels at the baseline were not different in both groups (Table 2). After the citrate infusion was finished, the Ca^2+^ concentrations were slightly decreased to 1.03 ± 0.12 and 0.86 ± 0.22 mmol/L in the ALF and ACLF groups, respectively. However, the Ca^2+^ level tended to be lower in the ACLF than the ALF patients during citrate infusion. After the citrate infusion was stopped (after 120 min), the Ca^2+^ concentrations gradually increased to a baseline of 1.12 ± 0.11 and 0.98 ± 0.18 mmol/L in the ALF and ACLF groups, respectively, (Fig. 1B).

The ACLF patients tended to have lower Mg^2+^ levels than the ALF patients, but both reached their minimum concentration at the end of the citrate infusion (120 min) of 0.51 ± 0.1 and 0.36 ± 0.07 mmol/L in the ALF and ACLF patients, respectively. After the citrate infusion was stopped, the Mg^2+^ concentration was gradually increased to the baseline in both groups (0.60 ± 0.08 mmol/L vs. 0.46 ± 0.07 mmol/L in the ALF and ACLF groups, respectively) (Fig. 1C).

### Metabolic effect

Metabolic profiles were comparable at baseline between the two groups (Table 2). During citrate infusion, the pH slightly decreased in both groups and gradually increased after citrate infusion was stopped (Fig. 1D). However, one patient in the ACLF group developed severe metabolic acidosis (pH < 7.2) during citrate infusion. The pH did not improve after infusion and so it could likely be explained from the underlying disease of this patient and citrate accumulation due to liver impairment. No incidence of metabolic alkalosis occurred during citrate infusion in both groups.

### Citrate accumulation

**Figure 2:**
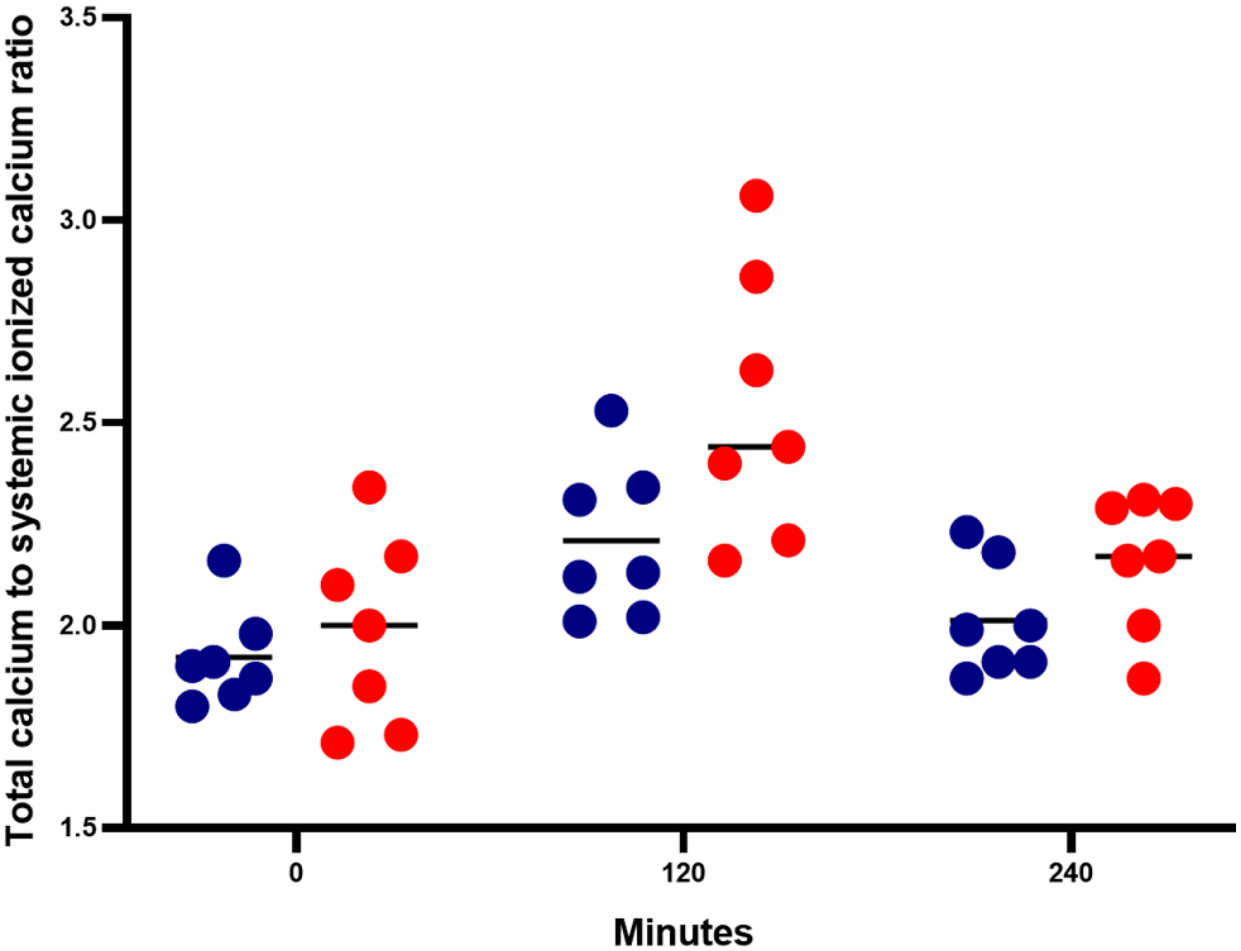
Comparison of the total calcium: systemic ionized calcium ratio between ALF (blue circles) and ACLF (red circles) at baseline, 120 min (end of 120-min citrate infusion), and 240 min (120-min after citrate infusion stopped).

Given the risk of citrate toxicity, we monitored the Ca_tot_/Ca^2+^ ratio every 120 min. The average baseline Ca_tot_/Ca^2+^ ratio was 1.92 ± 0.12 vs. 1.99 ± 0.24 in the ALF and ACLF groups, respectively. However, after citrate infusion for 120 min, both the ALF and ACLF groups showed increased Ca_tot_/Ca^2+^ ratios, and this was higher in the ACLF than the ALF group (2.54 ± 0.33 vs. 2.21 ± 0.19, respectively). In the ALF group, one patient showed a Ca_tot_/Ca^2+^ ratio of more than 2.5 (level of 2.53) without metabolic acidosis. However, in the ACLF group, three patients had a Ca_tot_/Ca^2+^ ratio of > 2.5 (2.63, 2.86, and 3.06) and two of them had metabolic acidosis, confirming the diagnosis of citrate accumulation. After stopping citrate infusion all patients had a normalized Ca_tot_/Ca^2+^ ratio (2.01 ± 0.14 and 2.16 ± 0.17 in the ALF and ACLF group, respectively) and no evidence of citrate accumulation was found (Fig. 2).

## Discussion

This study is the first to demonstrate citrate PK and clinical outcomes in critically ill ALF and ACLF patients receiving CRRT. Interestingly, the citrate PK were different to the results of a previous study^13^. Firstly, compared with the previous reported citrate clearance of 648.0 ± 347.0 mL/min in critically ill patients with CRRT^13^, the citrate clearance was impaired in both the ALF and ACLF patients at 152.5 ± 50.9 and 195.6 ± 174.3 mL/min, respectively. This reduced total citrate clearance was clinically confirmed by the decrease in pH and Ca^2+^ concentration during citrate infusion. Secondly, we found an increased Ca_tot_/Ca^2+^ ratio in both the ALF and ACLF groups (2.21 ± 0.19 and 2.54 ± 0.33, respectively), and two ACLF patients (28.5%) were diagnosed with citrate accumulation.

Both ALF and ACLF were common in critically ill patients receiving CRRT, and is associated with an increased risk of coagulopathy, thrombocytopenia, and bleeding^17^. To this concern, the Kidney Disease Proving Global Outcomes (KDIGO) Clinical Practice Guideline for AKI^1^ noted that severe liver failure is a major contraindication of RCA due to the risk of citrate toxicity. Our results were aligned with a previous study that citrate metabolism was decrease by approximately 50% in ALF without AKI^9^. However, compared to the previous studies of citrate PK and metabolism in CRRT^13,14^, we found the citrate clearance in ALF and ACLF patients was decreased by approximately 50% compared to that for cirrhotic patients (340 ± 185 mL/min) and approximately 75% compared to other critically ill patients (686.6 ± 353.6 mL/min). Other PK components, compared to two other studies^13,14^, are summarized in Supplementary Table 3.

Typically, a filter clearance (Cl_filter_) can be calculated by 2 methods. The first method uses sieving coefficient (SC) that is calculated by plasma and hemofiltrate concentrations as SC = C *UF*/C *Plasma*. So, the filter clearance can be determined as Cl_filter_ = SC x ultrafiltration rate. The second one as we applied in our study utilizes plasma concentrations before entering (C*in*) and after passing filter (C*out*) as Cl_filter_ = blood flow rate × ((C*in* – C*out*)/C*in*)^18,19^. Welte and colleagues performed the trimethoprim/sulfametrole in critically ill patients on CRRT. The Cl_filter_ in this study was calculated by two previously described methods. The results showed that the Cl_filter_ of trimethoprim (1.8 vs 1.4 L/h) and sulfametrole (1.1 vs 1.1 L/h) from both calculation methods were similar^20^. Moreover, the second method has been used in other previously published PK studies in patients receiving RRT with other drugs^21,22^.

To our knowledge, citrate PK studies in patients receiving CRRT were very limited. Zheng and colleagues demonstrated citrate PK in critically ill AKI patients receiving CRRT^13^. They also calculated the filter clearance using C*in* and C*out* (second method). From aforementioned reasons and to compare the body citrate clearance (the total citrate clearance subtracting by filter citrate clearance) from Zheng’s and our results as shown in Supplementary Table 3, we decided to use the same Cl_filter_ equation as shown in Zheng’s.

Citrate is removed by CRRT and its SC was reported as approximately 1 in previous literature^23,24^. However, PK changes such as volume overload, hypoalbuminemia and wide variation in protein binding are commonly seen in critically ill patients. These would affect citrate removal parameters such as SC and Cl_filter_ from CRRT in individual patients^25,26^. To accurately demonstrate the Cl_filter_ in those vulnerable patients, we decided to collect blood samples from C*in* and C*out* in for clearance calculation in our study.

During citrate infusion, both systemic Ca^2+^ and Mg^2+^ are chelated by the citrate, resulting in ionized hypocalcemia and ionized hypomagnesemia^27^. This study revealed a decreased level of both ionized hypocalcemia and ionized hypomagnesemia, which reached a minimum after 120 min (end of the citrate infusion) in both groups. However, the ACLF group had a lower ionized hypocalcemia and a lower ionized hypomagnesemia than the ALF group, which resulted from the decreased citrate clearance. Given the metabolic disturbance effect of citrate in this study, the pH tended to be shifted to an acidotic range during citrate infusion and the ACLF group had a lower average pH than the ALF group. This effect could also be explained by the impairment of the citric acid cycle production of bicarbonate. These effects were improved after stopping citrate infusion with a shift from acidification towards alkalization.

Another concern about using RCA in liver failure patients is the potential for citrate accumulation. This study reported a higher Ca_tot_/Ca^2+^ ratio (> 2.5) and two ACLF patients (28.5%) showed evidence of citrate accumulation during citrate infusion. Although, many studies have performed CRRT using RCA and reported a low incidence of citrate accumulation via intensive monitoring of the calcium and acid–base status^17,28–30^, our study showed the potential for citrate accumulation in patients with liver impairment.

Interestingly, our study found that the body citrate clearance in ALF was lower than in ACLF group. There might be explained by higher severity of liver injury and lower metabolic capacities to detoxify albumin bound toxin in ALF than in ACLF^31,32^. We showed that high incidence of low systemic Ca^2+^, high Ca_tot_/Ca^2+^ ratio, and high rate of citrate accumulation in ACLF group. This result might cause by the difference in baseline characteristics and laboratory between both groups. In our study, there was lower baseline systemic Ca^2+^ in ACLF group compared to ALF group (1.08 ± 0.22 mmol/L vs 1.18 ± 0.10 mmol/L) (Table 2, Fig. 1B). The systemic Ca^2+^ was used as a marker to of citrate related complication. Many studies of RCA among liver disease used the high calcium infusion rate or calcium-containing dialysate to avoid systemic hypocalcemia and high Ca_tot_/Ca^2+^ ratio^17,33^. However, systemic ionized hypocalcemia among critically ill patients is multifactorial cause included abnormal parathyroid hormone secretion, vitamin D deficiency, transfusion of citrated, the effect of circulating catecholamines, and disease severity^34,35^. Finally, the difference of clinical citrate related metabolic effect between both groups could not absolutely concluded due to the short citrate infusion times and the limited number of patients.

Of note, there was a different citrate dosing regimen between^13^ and our study. We applied continuous citrate infusion at 3 mmol/L as a total dose of 39.9 mmol in each case, with an average AUC_0−t_ of 124.4 ± 43.9 and 113.9 ± 73.3 in the ALF and ACLF groups, respectively. Whereas, the previous study^13^ utilized a total dose of 63.7 ± 9.1 mmol, with an average AUC_0−t_ of 79.7 ± 95.5 mmol that was comparable to healthy patients without AKI. To compare the AUC from these studies, we normalized the AUC by the total dose of 63.7 mmol. The normalized AUCs from our two liver impairment groups, based on the citrate dose from the previous study^13^, were 198.60 and 181.76 mmol*min/L in the ALF and ACLF groups, respectively. Clearly, the normalized AUC from the ALF and ACLF groups in this study were considerably (2.28- to 2.49-fold) higher than in the previous study^13^. As aforementioned, the clinical effects of citrate toxicity were revealed in some patients from our study, and so we would recommend that the citrate dosing regimens should be reduced at least two-fold to a citrate infusion dose of 1.5 mmol/L, to avoid citrate accumulation and toxicity.

This study had several strengths. This is the first reported clinical PK and metabolism of citrate in decompensated liver failure receiving CRRT, which is the knowledge gap of this area. Based on reported citrate PK changes from our study, we suggest that RCA use in these liver injury patients should be closely monitored to avoid citrate-related complications, such as citrate accumulation or severe hypocalcemia. The results of our study can be generalized to the real-life practice as the critical conditions, AKI undergoing CRRT and liver injury, are commonly seen in ICU settings.

Our study had several limitations. At first, we used only CVVH treatment for CRRT, while there is other CRRT modalities, such as CVVHD and CVVHDF, used in different clinical scenarios that might give different results. Secondly, the citrate infusion time was only 120 min and so we could not follow the clinical outcomes of long-term metabolic and citrate toxicity. A clinical study with a long-term follow up timeframe would be needed. Thirdly, our baseline citrate concentration did not start at zero mmol, which might affect the exogenous citrate level from the blood product and the impairment of citrate clearance in these patients. Fourthly, due to limited number of patients enrolled in this study, the population PK modelling and the correlation of each covariation and clinical parameters to citrate PK could not be performed. Further study with higher numbers of patients is absolutely needed. Finally, the definitions of ALF and ACLF in our study derived from The Asian Pacific Association for the Study of the Liver (APASL) consensus, which based on Asia–Pacific region. Therefore, our results may not be generalizable among other ALF and ACLF definitions such as Europe and Western region, which the definitions derived from the European Association for the Study of the Liver (EASL)^36^.

## Conclusions

Our study demonstrated that citrate clearance in critically ill patients with decompensated liver failure was significantly impaired by 50% and 75% compared to compensated cirrhotic and other critically ill patients, respectively. Despite the recent data on the feasibility of citrate in these patients, we still emphasize using a lower dose of citrate with intensive monitoring of calcium, Ca^2+^, and electrolyte concentrations to prevent citrate-related complications. Further studies needed to be provided for RCA use in liver failure patients.

## Supplementary Information

Supplementary Figure 1.

Supplementary Table 1.

Supplementary Table 2.

Supplementary Table 3.

## Data Availability

On reasonable request, data from this study are available from the corresponding author.
