# Supplementary material for: Citrate pharmacokinetics in critically ill liver failure patients receiving CRRT
Source: Sci Rep. 2022 Feb 2;12:1815. doi: 10.1038/s41598-022-05867-8 (PMC8810887; doi:10.1038/s41598-022-05867-8)
Supplement: Supplementary file 1 — Supplementary Figure 1. [file 41598_2022_5867_MOESM1_ESM.docx]

**Supplement Figure 1** **Blood sample Timelines**


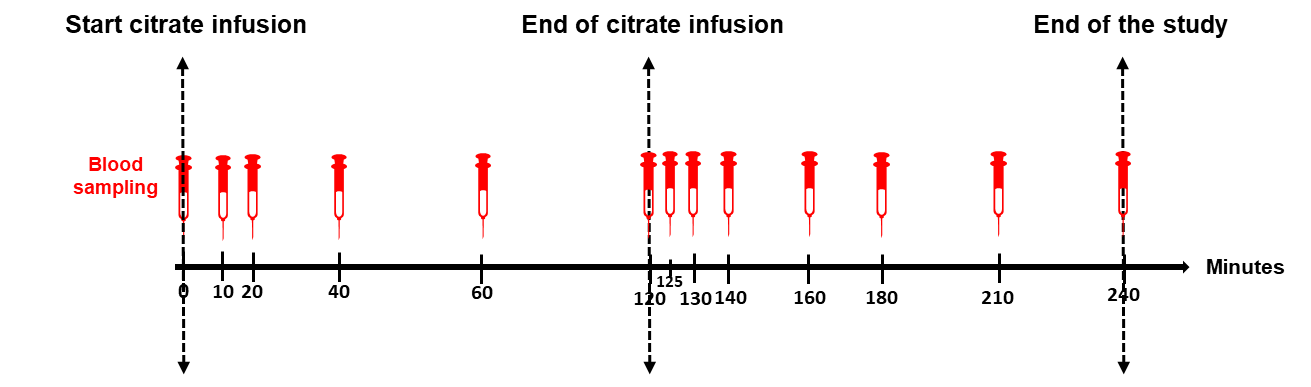


Citra-HF-Pre (Nordic Medcom, Finland) was used as the pre-dilution replacement fluid for administration of isotonic citrate solution for 120 min. The citrate dose was prescribed at 3 mmol/L by adjusting the blood flow rate and pre-filter replacement fluid rate depending on the bodyweight of each patient.

The red syringe shows blood sampling time points from the pre-filter port at the timelines for citrate concentration, blood gas, electrolyte, calcium, magnesium, Ca^2+^, and Mg^2+^.

In addition, 10 min after the end of citrate infusion (130 min), blood samples were taken simultaneously from both the pre-filter and post-filter
