# Supplementary material for: Citrate pharmacokinetics in critically ill liver failure patients receiving CRRT
Source: Sci Rep. 2022 Feb 2;12:1815. doi: 10.1038/s41598-022-05867-8 (PMC8810887; doi:10.1038/s41598-022-05867-8)
Supplement: Supplementary file 2 — Supplementary Table 1. [file 41598_2022_5867_MOESM2_ESM.docx]

**Supplementary Table 1 CRRT Protocol**

|  | | iCa^2+^ value | 0.8–0.9 mmol/L | 0.9–1.0 mmol/L | 1.0–1.1 mmol/L |
| --- | --- | --- | --- | --- | --- |
| BFR (mL/min) | **Pre-filter replacement rate (mL/h)** | **BW (kg)** | **Calcium rate (mL/h)** | | |
| 110 | 1500 | < 60 | 11 | 9 | 7 |
| 140 | 1900 | 60-70 | 15 | 11 | 9.5 |
| 160 | 2100 | > 70 | 16.5 | 12.5 | 10.5 |
| 180 | 2400 | > 80 | 18 | 14 | 12 |

Abbreviations: BFR, blood flow rate; BW, body weight; iCa, ionized calcium
