# Supplementary material for: Citrate pharmacokinetics in critically ill liver failure patients receiving CRRT
Source: Sci Rep. 2022 Feb 2;12:1815. doi: 10.1038/s41598-022-05867-8 (PMC8810887; doi:10.1038/s41598-022-05867-8)
Supplement: Supplementary file 3 — Supplementary Table 2. [file 41598_2022_5867_MOESM3_ESM.docx]

**Supplementary Table 2 Patients’ CRRT Prescription**

| Patient | BW (kg) | BFR (mL/min) | Pre-dilution replacement rate (mL/h) | Calcium rate (mL/h) |
| --- | --- | --- | --- | --- |
| 1 | 50 | 110 | 1500 | 7 |
| 2 | 40.5 | 110 | 1500 | 7 |
| 3 | 60 | 110 | 1500 | 7 |
| 4 | 58 | 110 | 1500 | 7 |
| 5 | 47 | 110 | 1500 | 7 |
| 6 | 45 | 110 | 1500 | 7 |
| 7 | 70 | 140 | 1900 | 9.5 |
| 8 | 48.5 | 110 | 1500 | 9 |
| 9 | 54 | 110 | 1500 | 7 |
| 10 | 62 | 140 | 1900 | 9.5 |
| 11 | 65 | 140 | 1900 | 9.5 |
| 12 | 74 | 160 | 2100 | 10.5 |
| 13 | 53 | 110 | 1500 | 7 |
| 14 | 69 | 140 | 1900 | 15 |

Abbreviations: BFR, blood flow rate; BW, body weight
