# Supplementary material for: Citrate pharmacokinetics in critically ill liver failure patients receiving CRRT
Source: Sci Rep. 2022 Feb 2;12:1815. doi: 10.1038/s41598-022-05867-8 (PMC8810887; doi:10.1038/s41598-022-05867-8)
Supplement: Supplementary file 4 — Supplementary Table 3. [file 41598_2022_5867_MOESM4_ESM.docx]

**Supplementary Table 3 Comparison of Citrate PK studies**

|  | Our study | | Kramer et al* | Zheng et al** |
| --- | --- | --- | --- | --- |
| **Patients’ characteristics** | | | | |
| **Population** | Critically ill ALF patients | Critically ill ACLF patients | Critically ill cirrhosis patients | Critically ill patients |
| **Number of patients** | 7 | 7 | 16 | 12 |
| **Age (y)** | 59.9 ± 12.8 | 68.1 ± 19.8 | 55 ± 10 | 52.7 ± 16.9 |
| **APACHE II score** | 19.6 ± 4.1 | 24.0 ± 5.6 | 18 ± 8 | NA |
| **SOFA scores** | 13.7 ± 3.7 | 15.6 ± 3.5 | NA | 10.7 ± 4.2 |
| **Total bilirubin (mg/dL)** | 19.5 ± 8.9 | 11.1 ± 5.1 | 13.2 ± 13.3 | 2.05 ± 2.97 |
| **AST (U/L)** | 128 (50,355) | 165 (58,614) | 38 ± 24 | 41.5 (28.5,164.5) |
| **ALT (U/L)** | 120 (23,352) | 112 (21,224) | 20 ± 18 | 26 (10.5,164.5) |
| **Citrate PKs:** | | | | |
| **AUC_0-t_ (mmol.min/L)** | 124.4 ± 43.9 | 113.9 ± 73.3 | NA | 69.9 ± 66.6 |
| **AUC_0-inf_ (mmol.min/L)** | 267.2 ± 111.7 | 372.6 ± 399.3 | 282 ± 130 | 87.5 ± 95.5 |
| **T_max_ (min)** | 100.0 ± 60.0 | 113.8 ± 32.8 | 115 ± 12 | 106.6 ± 21.7 |
| **V_d_ (L)** | 45.6 ± 8.0 | 58.2 ± 49.7 | 27 ± 9 | 50.6 ± 21.7 |
| **Cl _body_ (mLmin)** | 152.5 ± 50.9 | 195.6 ± 174.3 | 340 ± 185 | 686.6 ± 353.6 |
| **C _baseline_ (mmol/L)** | 0.24 ± 0.12 | 0.21 ± 0.12 | 0.51 ± 0.13 | 0.02 ± 0.04 |
| **C _max_ mmol/L)** | 0.76 ± 0.27 | 0.72 ± 0.44 | 1.6 ± 0.5 | 0.56 ± 0.45 |
| **Total dose (mmol)** | 39.9 | 39.9 | 77 ± 21 | 57.1 ± 10.5 |

Abbreviations: AUC, area under the concentration time curve; T_max_, time to maximum concentration; V_d_, volume of distribution, Cl_body_, citrate clearance by body; C_baseline_, baseline citrate concentration; C_max_, maximum citrate concentration

*Data from Kramer L, et al. Citrate pharmacokinetics and metabolism in cirrhotic and noncirrhotic critically ill patients. *Crit Care Med*. 2003;31(10):2450-2455. doi:10.1097/01.CCM.0000084871.76568.E6

**Data from Zheng Y, et al. Citrate Pharmacokinetics in Critically Ill Patients with Acute Kidney Injury. *PLoS One*. 2013;8(6):e65992. Published 2013 Jun 18. doi:10.1371/journal.pone.0065992
